# Athlete Atypicity on the Edge of Human Achievement: Performances Stagnate after the Last Peak, in 1988

**DOI:** 10.1371/journal.pone.0008800

**Published:** 2010-01-20

**Authors:** Geoffroy Berthelot, Muriel Tafflet, Nour El Helou, Stéphane Len, Sylvie Escolano, Marion Guillaume, Hala Nassif, Julien Tolaïni, Valérie Thibault, François Denis Desgorces, Olivier Hermine, Jean-François Toussaint

**Affiliations:** 1 IRMES, Insep, Paris, France; 2 INSERM, U970, Paris, France; 3 Paris Descartes University, Faculty of Sciences, Paris, France; 4 Service d'hématologie Hôpital Necker and CNRS UMR 8147, Paris, France; 5 Centre d'Investigation de la Médecine du Sport (CIMS), Hôtel-Dieu, Assistance Publique - Hôpitaux de Paris, Paris, France; Pennington Biomedical Research Center, United States of America

## Abstract

The growth law for the development of top athletes performances remains unknown in quantifiable sport events. Here we present a growth model for 41351 best performers from 70 track and field (T&F) and swimming events and detail their characteristics over the modern Olympic era. We show that 64% of T&F events no longer improved since 1993, while 47% of swimming events stagnated after 1990, prior to a second progression step starting in 2000. Since then, 100% of swimming events continued to progress.

We also provide a measurement of the atypicity for the 3919 best performances (BP) of each year in every event. The secular evolution of this parameter for T&F reveals four peaks; the most recent (1988) followed by a major stagnation. This last peak may correspond to the most recent successful attempt to push forward human physiological limits. No atypicity trend is detected in swimming. The upcoming rarefaction of new records in sport may be delayed by technological innovations, themselves depending upon economical constraints.

## Introduction

Sport performances may cease to improve during the XXI^th^ century, possibly due to physiological limits [1] and interactions between genomic [2] and environmental parameters [3]. The progression of top athletes' performances remains unknown for quantifiable sport events. We hypothesize that such an evolution also mirrors our social and historical development [4] and symbolize our quest for the Citius. Modelling this progression enables us to identify the underlying trends and behaviours in sports and history on a world scale. After the publication of initial mathematical models [5], [6], world records (WR) were shown to follow a piecewise exponential model [1] over the modern Olympic era (1896–2007). The present study is based on the analysis of a large scope of sport performances as an indicator of our species' physiological maxima. It encloses the human physical potential and may be seen as a complement to direct laboratory measurements on a sample of elite athletes [7].

In this study we analyse the single best result of the top 10 world performers every year from two major olympic disciplines (track & field – T&F – and swimming) in order to establish their law of progression after one century of sport development. We also introduce the concept of “*atypicity*”, defined as the singularity trait of a given performance, and we measure its trend in the sport law of progression. Atypicity is seen in all systems. A previous study ranked T&F WR using extreme value theory [8] but did not investigate their temporal tendencies or the relationship between the best performer and the other athletes. A closer observation allows for scoring all performances using 3 statistical descriptors, characterizing atypicity, and providing a reliable trend of the *avant-garde* of human performers.

## Materials and Methods

We collected the single best result of the top 10 world performers every year in 70 events from the major two quantifiable Olympic disciplines: 36 T&F events over the modern Olympic era (1891–2008) and 34 swimming events over the 1963–2008 period [9]–[11]. A total number of 41351 performances including 3919 Best Performances (BP) were gathered.

### Growth Law

The law of progression after one century of sport development is modelled using a Gompertz function, widely used in biology [12], economic dynamics [13] or technology diffusion [14]–[15]. The physiological limit for each event was given by computing the year corresponding to 99.95% (1/2000^th^) of the estimated asymptotic value (Table S1). Events presenting a limit before 2008 were considered as “halted” events. In addition, we conducted a residual analysis (Materials and Methods S1) and determined the Most Recent Change Of Incline (MRCOI, Materials and Methods S1) for swimming events.

### Descriptive Analysis

Descriptive statistics were also conducted to assess the impact of the Olympic Games on performances every four years and to measure the yearly variation between each performer using the yearly mean relative performance improvement 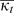 and the coefficient of variation 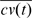.

### Measurement of the Atypicity

We finally focused on the *atypicity* of each BP using a set of specific descriptors: *d_1_* measured its relative distance to all other performances during the year, *d_2_* was the “*durability*” of a BP over the years before it is beaten by another performance and *d_3_* characterized the weight of each BP over all other performances for each event during the Olympic era. The highest 5% values of each descriptor are selected and studied for both disciplines. We define “*Atypicity*” 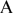 as the distance from each BP to the origin of the descriptors' uniformized Euclidian space.

## Results

### Growth Law

The average adjusted R^2^ for all 147 historical curves is 0.68±0.22 (mean ±standard deviation).

Among T&F events, 63.9% no longer progress (77.8% of the 18 women events; 50% of the 18 men events). The average year for the detected dates of halt in performance is 1992.8±7.9 (1991.8±8.0 for women; 1994.8±7.9 for men). Dates of halt range from 1980.9 (1500m women) to 2007.1 (triple jump men, Table S1).

The changes of incline in the T&F curve are mostly related to World Wars I and II (WWI, WWII) or changes in event timing methods, improving measurement accuracy, which is especially important over short events. However, in the last twenty years no rule alterations or technological improvements were made that could have resulted in a new curve.

Thirteen T&F events still progress (4 women's and 9 men's), nine of which are middle and long distance races.

Among swimming events, 100% still progress. The average MRCOI in swimming is 1997.2±2.7 and the peak appears in 2000. Specifically, the mean MRCOI for fly and breast swim styles is 1994.4±2.5 while the mean MRCOI for freestyle and back is 1998.1±2.1. Prior to the MRCOI, progression in 16 (47.1%) of the 34 events had halted: 8 women and 8 men events. Average year of halt is 1990±4.4 (1988.6±4.8 for women, 1991.5±3.6 for men).

### Descriptive Analysis

The impact of WWI and WWII for T&F events over 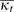 are computed: a regression of −0.44% of the mean performances within a six-year time span from 1913 to 1918 is observed for WWI; and −0.45% in a 6-year time span from 1940 to 1945 for WWII.

Average Olympic periodicity is measured with a mean increase of T&F performances of 0.99%±0.56 for Olympic year *t*, −0.32%±0.49 for *t+1*, 0.48%±0.45 for *t+2* and 0.37%±0.41 for *t+3*.

The mean yearly coefficient of variation 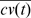 regresses over the century and range from 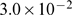 (1891) to 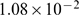 (2008) for T&F, and 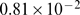 (2008) for swimming.

### Atypical Performances Measured through 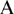


All of the descriptors' distributions for T&F and swimming reveal a right-skewed profile: outlying performances are located in right tails.

Secular evolution of the highest 5% values for each descriptor reveals 4 historical peaks in T&F: 1943 (14 cases), 1988 (13 cases), 1993 (7 cases) and 1998 (16 cases). Evolution of the highest 5% values remains steady in swimming over the period 1982–2008 except in 1994 where 17 cases are spotted.

The secular trend is given for each descriptor: descriptor *d_1_* decreases throughout the century, reaching a stable value in the 1980's, showing that the distance from the best performer to the others is decreasing, except in the WWII era's peak in 1943. Life expectancy or “*durability*” of a BP (*d_2_*) is stable over the century, with high values appearing in the 1980's in T&F. The most “*durable*” BPs were established during this period. The deviation produced by each BP over all other performances of the same event (*d_3_*) is stable over the century. High values are found during the following years: 1943 (*d_1_*, *d_3_*), 1988 (*d_2_*), 1993 (*d_3_*) for T&F and 1994 (*d_1_*, *d_4_*) for swimming.

Historical evolution of yearly “Atypicity” (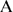) shows 4 peaks in T&F: 1916, 1943, 1968 and 1988 of amplitudes 0.39, 0.46, 0.31 and 0.46 respectively. Four corresponding cycles were found with durations of: 21, 28, 23 and 27 years accordingly.

There are no significant variations of 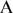 in swimming between 1982 and 2008.

## Discussion

### Progression Law of the “10 Best” and Descriptive Statistics

Analysis of top athletes' performances suggests that the progression of human performances may reach its limit soon [1], [3], [5], [6], [8]. WR were shown to follow a similar development [1], [3] that also highlighted that 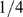 of T&F WR had reached their limit in 2008. We here introduce a new tool to describe the physiological dynamics of elite sport performances by modelling growth curves over a large data sample in 34 events since 1963 and 36 events over 118 years.

**Figure 1:**
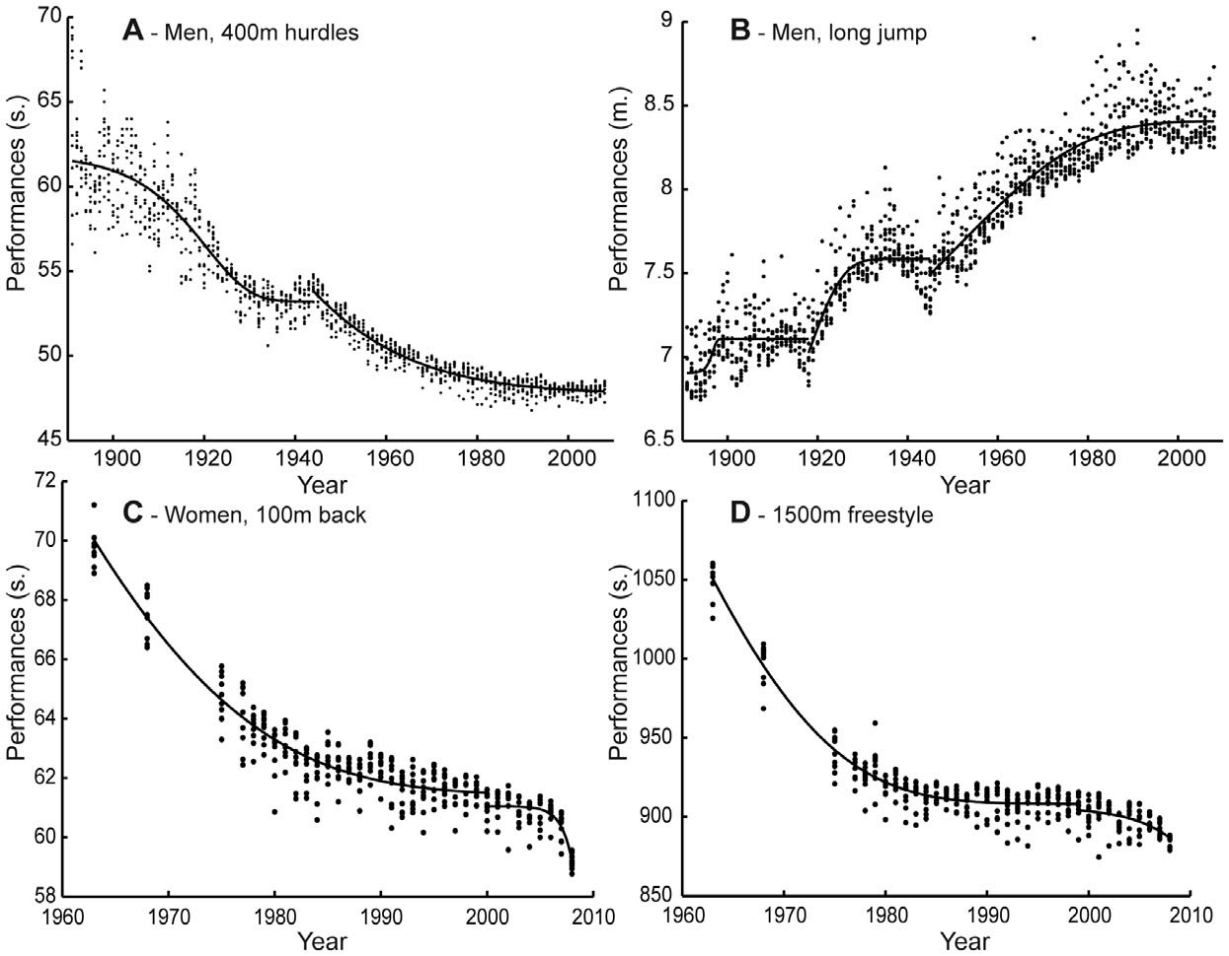
Model fitting on 4 events. **A**. Men 400m hurdles (T&F) fitting (R^2^i = 0.77; R^2^_ii_ = 0.91), progressing event. **B**. Men long jump (T&F) (R^2^_i_ = 0.22; R^2^_ii_ = 0.55; R^2^_iii_ = 0.81), “*halted*” event since 2001.5 (Table S1). **C**. Women 100m back (swimming) (R^2^_i_ = 0.90; R^2^_ii_ = 0.63). **D**. Men 1500m freestyle (swimming) (R^2^_i_ = 0.92; R^2^_ii_ = 0.31). Fewer data are available in swimming (C, D) between 1963 and 1977. Prior to the MRCOI, C was in progression and D was a “halted” event since 1991.9. A new progression trend appears after the MRCOI (2000 (C)/1999 (D)) for swimming events, as a result of the introduction of swimsuits.

The two studied disciplines show different progression schemes: most (63.9%) of T&F events have stopped progressing since 1993±8 years while all swimming events were progressing until 2009 (Fig. 1). This halt occurred 34 years earlier than the estimated stagnation of half of the WR in 5 Olympic disciplines [1]; it may reveal that most of T&F athletes are already beyond the edge of stagnation. Both genders present a slightly different evolution in T&F events, suggesting male events still have some potential reserve whereas the majority of women events (77.8%) has stopped progressing since 1992±8 years. Women may have reached their limits before men, despite a later entry into Olympic competition [16]. Positive values of the mean relative performance improvement are also attributable to the recent introduction of women events (pole vault, marathon, triple jump and 1500m).

The analysis of residuals (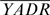) in T&F events reveals the impact of both world wars on performance development (Fig. S1). Following the Cold War period, a large regression (3.07%) is noticed in 4 events (shot put women, discus throw women, high jump men, long jump men, Fig. S2). The fact that the major two powers were in a dense competition [4] may have lead to a transitory extra-improvement which disappeared shortly after the war. From that point on, physiological progression may be limited in a majority of T&F events, and performance will not increase until international instances or federations allow for major technological improvement [17], [18].

**Figure 2:**
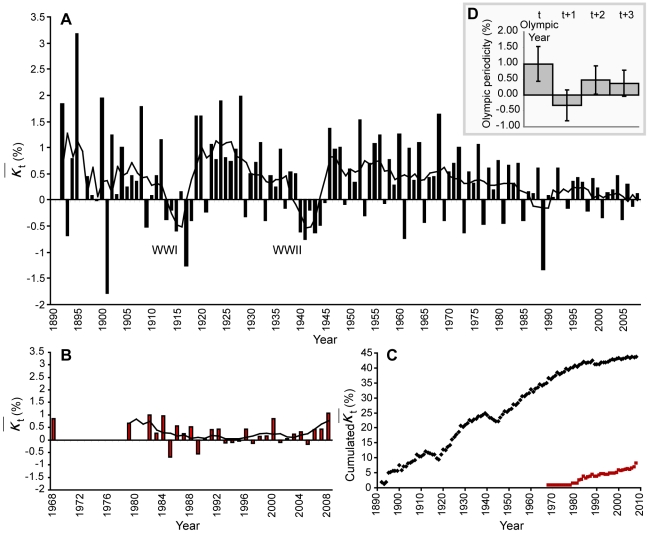
Evolution of performance improvement 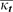 and Olympic periodicity. **A**. Evolution of 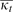 in T&F. **B**. Evolution of 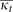 in swimming. **C**. Cumulative evolution of 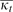 in T&F (black dots) and swimming (red squares). **D**. Average Olympic periodicity with standard deviation in T&F and swimming. Both 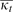 evolutions in T&F and swimming (A, B) are given with a 4 year smoothing average (black line). After a large period of performance improvement, hindered by the two World Wars, the development of performance slow down since 1960. A larger regression is observed in 1989. At this time, random anti-doping tests were established. The impact of the Olympic games, aka Olympic periodicity reveals that performances increase by 1% during an Olympic year (*t*), while they regress in the following year (*t+1*).

The recent progression period in swimming results owes much to the introduction of swimsuits (Fig. 2). This new technology, allowed by FINA in 1999, enhances hydrodynamic penetration and largely reduces drag forces [9], [19]. Results show a divergence in recent performance development between “*profiled*” styles (freestyle, back) and “*turbulent*” swim styles (fly, breast). Previous studies support this observation as they show that swimsuits may have more impact in breaststroke, the most turbulent style with the largest drag resistance to flow [20], [21]. The next step of performance development is seen in 2008 in relation to the introduction of second-generation swimsuits. This major performance increase culminated in the last Olympic Games (Beijing, 2008), where a single swimsuit was the common determinant of 96% of the 22 new WR, 95% of gold medals and 90% of all medals [22], [23].

Sport performance is an important indicator of the optimization of physiological functions, enzyme isoforms (actin, myosin, alpha-actinin [2]), energy use in aerobic and anaerobic metabolisms, oxygen transport and psychological resources that mobilize them [24]. Genes encoding each of these proteins or functions and their co-segregation or clustering are theoretically limited [25]. Applying the Gompertz model to the top performances of 70 sport events illustrates this concept through a description of performances evolving toward their limits. In fact, for a majority of T&F events, best performances show a secular progression hindered by the two WWs before reaching a phase of slower progression at the end of the XX^th^ century (Fig. 2). The evolution difference between the two disciplines is mostly based on technological improvement and rules alterations. In Beijing, T&F records were scarce compared to swimming: only 5 new WR were established with 3 assigned to the same exceptional athlete. The Berlin (2009 T&F World Championship: 3 WR) *vs.* Rome (2009 Swimming World Championship: 43 WR) competition confirmed the demonstration.

### Atypicity of the BP

The Gompertz model describes the evolution of the first 10 world performers in the past century (Fig. 1). However, some top performers stand apart from this group, suggesting their performances are remarkable. Such singular marks are in contrast with the tight evolution of the whole group. The stability of the yearly mean coefficient of variation (Fig. S3) is noticeable after 1960, suggesting that all athletes are benefiting from similar conditions for competitions (training facilities, technological and medical advances). The initial era (1891–1930) was a period during which social or economical conditions may have greatly differed between athletes. Later on, sport's internationalisation (1960–2008) has lead to the implementation of similar organizations, competition calendars and regulations among nations [4]. The continuous technological advances aiming to enhance performance were previously described by Robert Fogel as a “Techno-physiological evolution” [26].

**Figure 3:**
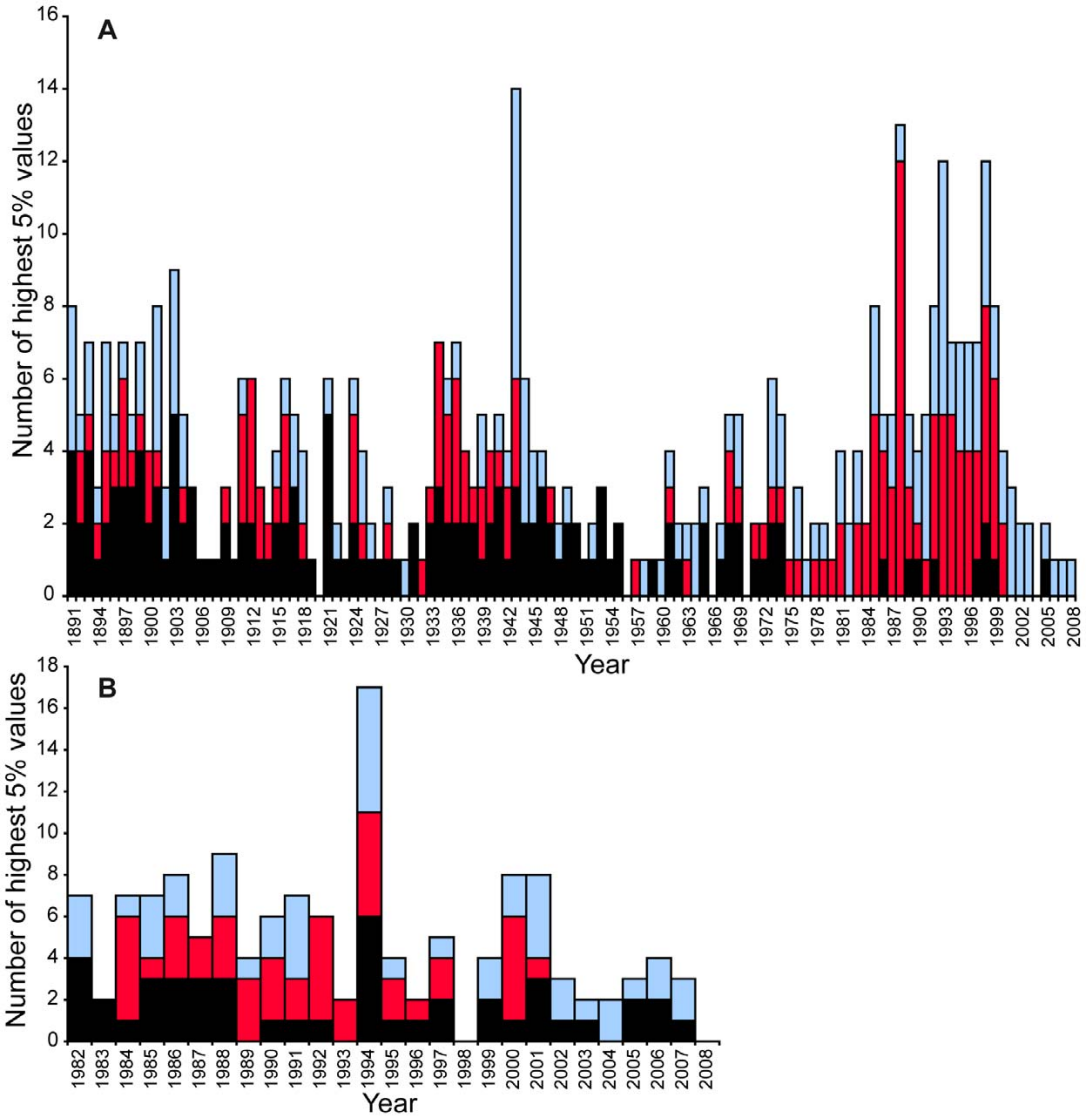
Number of highest 5% values by year and descriptor. **A**. T&F. **B**. swimming. The highest 5% values are gathered from each descriptor distribution in both disciplines: d_1_ (black), d_2_ (red), d_3_ (light blue).

The study of the highest 5% values of descriptors reveals 4 peaks for T&F and 1 peak for swimming (Fig. 3). Considering the post 1930 era in T&F, the 1943 peak is related to WWII. In this context, exceptional athletes are even more unique. The case of Fanny Blankers-Koen highlights that brilliant performers can score a maximum number of highest performances in such a period (4 gold medals in London Olympic games, 5 European titles and 16 WR in 8 events: 100 yards, 100m, 200m, 100m hurdles, high jump, long jump, pentathlon and 4*100m relay). This may be due to the presence of fewer skilled opponents.

The 1988 peak matches an Olympic year (Fig. 3). Eleven outstanding WR were beaten this year and 7 of them, in women events exclusively, still remain today. Only 3 were established in Seoul (200m women, 4*400m relay women, heptathlon). The others were established a few weeks earlier in Stara Zagora (BUL, 100m hurdles), Indianapolis (USA, 100m), Neubrandenburg (GDR, discus throw) and Leningrad (USSR, long jump). In this period, local competitions may also have had fewer monitoring procedures favouring illegal enhancement behaviours. Procedures for unexpected, out-of-competition anti-doping controls were officially approved one year later and 1988 can be considered as the T&F golden year of exceptional marks, which subsequently resulted in a large stagnation in the women's events.

Outstanding BP values of the 1993 peak do not correspond to an Olympic year, but may be linked to a generation of exceptional performers. This year, Chinese women athletes achieved exceptional performances in T&F with 33% of the BP, 33% of the second performances and 39% of the third performances. These ratios have never been equalled by China since then. On 1993 at the Chinese National Games in Beijing, 5 Chinese women athletes have beaten the 3000m WR, a singular moment in T&F history. A similar occurrence was only observed on the 26^th^ of July 1976 in Montreal, when 6 women athletes have beaten the WR in the same 800m race. All were East European athletes (Union of Soviet Socialist Republics (USRR), German Democratic Republic (GDR) and Bulgaria).

In swimming, evolution of atypical performances peaked in 1994 (Fig. S4). This year, Chinese swimmers achieved exceptional performances, obtaining 64.7% of the women's BP. In the last 30 years, only the GDR has established such a supremacy, with a 70.6% ratio in 1983, and 64.7% in 1987.

**Figure 4:**
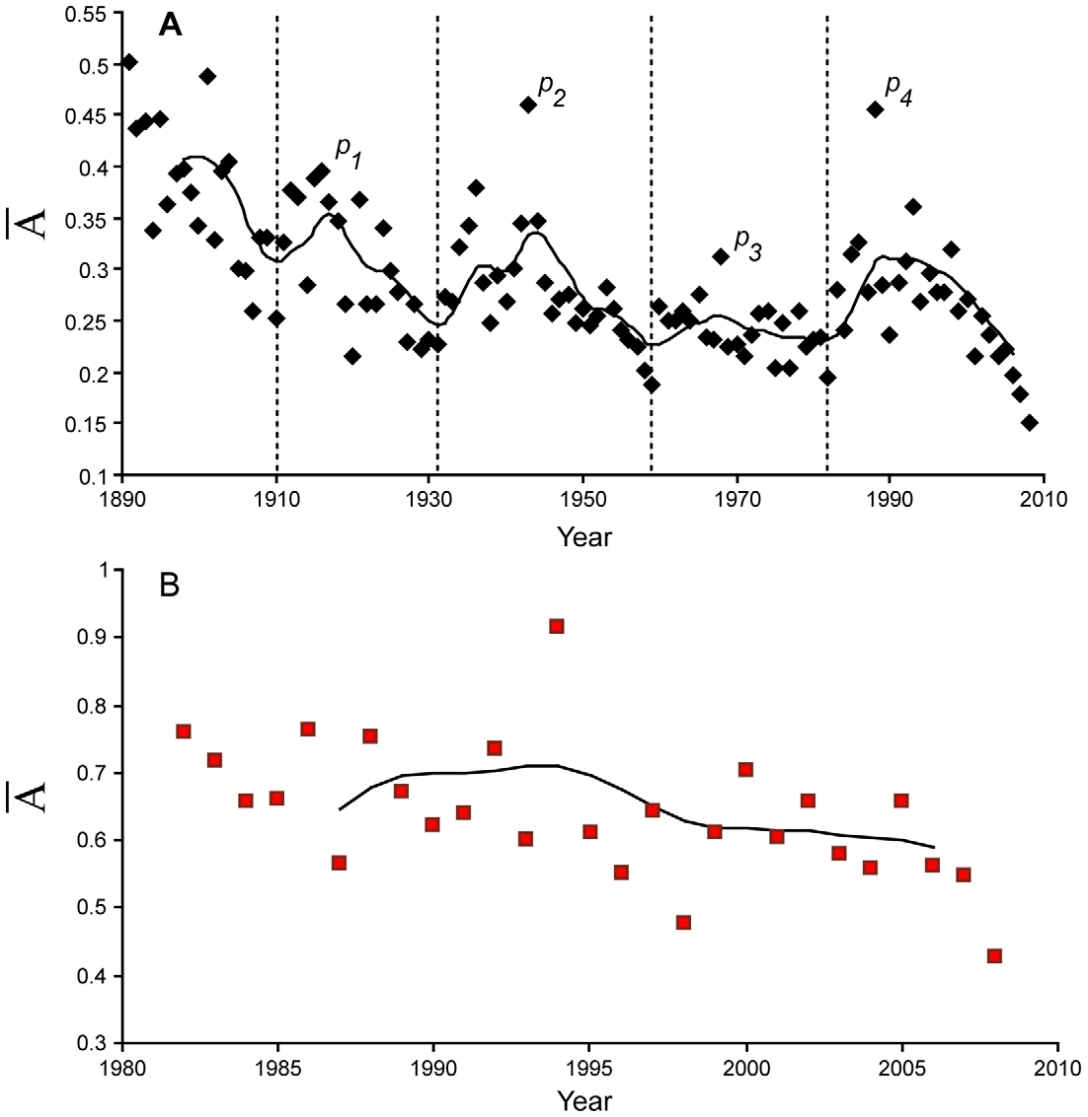
Average yearly 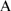 over time for each discipline. **A**. Secular evolution of 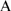 for T&F (black dots); **B**. Evolution of 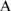 for swimming (red squares). Both evolutions are given with a 60 Hertz, second order low pass Butterworth filter. Besides the initial era (1890–1910), 4 peaks (*p_1_*–*p_4_*) appear in T&F. Swimming curve (B) does not show any trend over the years. The 1994 peak is mainly related to Chinese swimmers, who performed very high performances (also observable in 1993 (*p_4_*) in T&F).

Both disciplines present different distributions and spatial repartitions of descriptors (Fig. S4, Fig. S5). As a result, measure 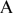 shows two different evolutions (Fig. 4). In T&F the first 2 peaks of 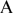 have the same significance as the first 2 peaks of the highest 5% values, corresponding to the world wars (Fig. 4). The third peak corresponds to the year of the Mexico Olympic Games (1968), during which numerous outstanding performances were established. This corresponds to the year with the highest number of WR [1]. The largest and final peak (1988) is followed by a major regression of performances and a “*halt*” of T&F progression. This peak may correspond to the last successful attempt of our species to push its physiological limits forward.

The observed discrepancy between athletes of different countries may be related to training protocols improvements. Russian training loads were increased three fold between 1968 and 1998, in speed and strength sports [27]. Most countries have followed such a trend in training protocols and volumes. Between 1950 and 1990, East European countries such as the GDR were involved in doping protocols [28] and may be responsible for many performances by undetected doped athletes [28]–[33]. As a more recent example, recombinant erythropoietin (EPO) was marketed in 1989 and the International Olympic Committee prohibited its use in sports in 1990 [34], [35]. Several studies quantified EPO's effect on performance by measuring maximal oxygen uptake (VO2max) and showed a 6.3 to 6.9% increase [36], [37]. The introduction of out-of-competition controls in 1988 may have led to a reduction of drugs use among athletes. The later introduction of World Doping Anti-Agency (WADA) in 1999 has led to the harmonization of rules and regulations governing the anti-doping struggle in 2004. These recent efforts in the fight against doping may have had an effect on the proportion of doped athletes in competition. This may have been the case in T&F where we see a general decline of performance after 1988 except in middle and long distance running races [7]. The same effect is observable in cycling [32]. In the present study, we cannot quantify the proportion of doped athletes so far. However, the analysis of this proportion among BP, using available lists from official sources [9], [38] might allow an estimation of the ratio of physiological *vs.* pharmacologically enhanced performances.

In swimming, 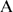 remains stable over the last quarter of century as swimsuits benefited most of the best performers (Fig. 4). Major advancements in sports heavily rely on materials developments. Duralumin, carbon, polyurethane (tartan track, first used in the 1968 Olympic Games, or swimsuits) granted temporary improvements in Olympic disciplines. Innovation is a driving force of performance development and technology will need continuous research development in order to produce materials with higher energetic efficiency and to defy performances stagnation. These new programs will also depend on economical incentives and balances [39].

### Conclusion

The Gompertz model of the first 10 world performers reveals that performances now stagnate in T&F while swimming still progressed until today. Atypicity quantifies the irregularity of the yearly best performer. The pinnacle of atypical T&F performances occurred in 1988, hastening the race toward human limits. This present halt of performances and the previously demonstrated stagnation of WR [1], [3] emphasize that our physiological evolution will remain limited in a majority of Olympic events. Present performances may now be enhanced through extremely exceptional individuals at the frontier of our genomic condition or with the artificial help of technology. However, the recent decision of FINA regarding the ban of specific swimsuits in 2010 will impact the future performance progression. If such a decision is confirmed, we may observe a rapid convergence toward the previously estimated physiological asymptotes. The limitation of artificial enhancements may drive performances back down to the physiological frontiers, which in turn depend upon growing economical or environmental constraints.

## Supporting Information

Table S1 Evolution of the 10 best performers in all 70 events. Women events (W) and Men events (M) are sorted by disciplines (T&F vs. swimming), and distances/type of efforts for T&F. All swimming events show progression after the MRCOI.
(0.10 MB DOC)

Materials and Methods S1 Model and descriptors (d1-d3) description. Description of the model and the Gompertz function. Description of the three statistical descriptors d1-d3.
(0.08 MB DOC)

Figure S1 Secular evolution of the yearly average distance of residuals (YADR). A. Secular evolution of YADR in T&F (gray bars, left ordinate) with the number of changes of inclines (red bars, right ordinate). B. Secular evolution of YADR in swimming (gray bars, left ordinate) with the number of MRCOI detected each year (red bars, right ordinate). The analysis of residuals of T&F events (A) presents several stages: two major deviations periods (Et1,Et2) consecutive to WWs and two more recent peaks (Et3,Et4) including the largest one (Et4). After the initial era (1890 to 1900), high positive deviations from the model are spotted during the following periods: 1908 to 1915 (Et1, peak in 1912: 1.31%), 1935 to 1942 (Et2, peak in 1939: 1.25%), 1953 to 1963 (Et3, peak in 1956: 1.04%), 1977 to 1991 (Et4, peak in 1988: 1.1%). Swimming YADR (B) presents numerous variations of low amplitudes (peak in 1984 at 0.6%). The major number of MRCOI detected in swimming is spotted in 1999 and 2000, contemporary to the introduction of swimsuits, allowed by the FINA in 1999.
(0.85 MB TIF)

Figure S2 Model fitting on 6 T&F. A. Men discus throw (T&F) (R2i = 0.85; R2ii = 0.83; R2iii = 0.94), “halted” in 1984.2. B. Women discus throw (T&F) (R2i = 0.88; R2ii = 0.92), “halted” in 1988.4. C. Women long jump (R2i = 0.70; R2ii = 0.90), “halted” in 1996.8. D. Women high jump (R2i = 0.78; R2ii = 0.95), “halted” in 1995.2. E. Men shot put (R2i = 0.43; R2ii = 0.80; R2iii = 0.93), “halted” in 1983.9. F. Women shot put (R2i = 0.86; R2ii = 0.90), “halted” in 1983.9.
(0.68 MB TIF)

Figure S3 Evolution of cv(t) factor in T&F and swimming. cv(t) values are decreasing over time in T&F (black dots); cv(t) values show no trend in swimming (red squares) on a shorter follow-up period. T&F cv(t) evolution is linked to historic events: after World War II values are getting tighter (<0.015) suggesting all performances are progressing in the same range.
(0.16 MB TIF)

Figure S4 Secular evolution of descriptors. Evolution is given in T&F (A, B, C, black dots) and swimming (D, E, F, red squares). Descriptor d2 presents a decreasing evolution from 2003 to 2008, which is related to time. It is assessed by T, measuring the number of performances still unbeaten (green squares) in both disciplines. High average values of T&F descriptors are spotted at d2: 1988, d3: 1993.
(0.37 MB TIF)

Figure S5 Spatial repartition and distributions of the 3 uniformized descriptors d1, d2, d3. Spatial repartition and distributions of the 3 uniformized descriptors d1, d2, d3 for T&F (A, black dots and bars) and swimming (B, red dots and bars). All distributions are unimodal with positive skew. For T&F: d'1 skew = 2.9; d'2 skew = 3.8; d'3 skew = 4.5; for swimming: d'1 skew = 0.6; d'2 skew = 3.6; d'3 skew = 1.7; p-value<0.001 for all (d'Agostino Skewness test, alternative hypothesis: positive skewness). Outliers are located in right tails.
(0.61 MB TIF)
