# Supplementary material for: Athlete Atypicity on the Edge of Human Achievement: Performances Stagnate after the Last Peak, in 1988
Source: PLoS One. 2010 Jan 20;5(1):e8800. doi: 10.1371/journal.pone.0008800 (PMC2808355; doi:10.1371/journal.pone.0008800)
Supplement: Table S1 — Evolution of the 10 best performers in all 70 events. Women events (W) and Men events (M) are sorted by disciplines (T&F vs. swimming), and distances/type of efforts for T&F. All swimming events show progression after the MRCOI. (0.10 MB DOC) [file pone.0008800.s001.doc]

| **Track & Field Events** | **Status (Actual)** | **Lock year** |
| --- | --- | --- |
| 100m (W) | Locked | 1991.8 |
| 100m Hurdles (W) | Progression |  |
| 200m (W) | Locked | 1982.4 |
| 400m (W) | Locked | 1985.9 |
| 400m Hurdles (W) | Locked | 1994.3 |
| 800m (W) | Locked | 1995.3 |
| 1500m (W) | Locked | 1980.9 |
| 3000m (W) | Locked | 1984.6 |
| 5000m (W) | Progression |  |
| 10000m (W) | Progression |  |
| Marathon (W) | Locked | 2004.7 |
| High Jump (W) | Locked | 1991.2 |
| Long Jump (W) | Locked | 1996.9 |
| Triple Jump (W) | Locked | 1995.3 |
| Pool Vault (W) | Locked | 2006.6 |
| Discus Throw (W) | Locked | 1988.5 |
| Shot Put (W) | Locked | 1984.0 |
| Hammer Throw (W) | Progression |  |
| 100m (M) | Progression |  |
| 110m Hurdles (M) | Locked | 1996.7 |
| 200m (M) | Progression |  |
| 400m (M) | Locked | 1989.1 |
| 400m Hurdles (M) | Progression |  |
| 800m (M) | Progression |  |
| 1500m (M) | Progression |  |
| 3000m Steeple (M) | Progression |  |
| 5000m (M) | Progression |  |
| 10000m (M) | Progression |  |
| Marathon (M) | Progression |  |
| High Jump (M) | Locked | 1994.8 |
| Long Jump (M) | Locked | 2001.6 |
| Triple Jump (M) | Locked | 2007.1 |
| Pool Vault (M) | Locked | 1994.9 |
| Discus Throw (M) | Locked | 1984.3 |
| Shot Put (M) | Locked | 1984.0 |
| Hammer Throw (M) | Locked | 2001.1 |
| **Swimming Events** | **Status (prior to MRCOI)** | **Lock year** |
| 50m FreeStyle (W) | Progression |  |
| 100m FreeStyle (W) | Progression |  |
| 100m Breast (W) | Progression |  |
| 100m Fly (W) | Locked | 1990.5 |
| 100m Back (W) | Progression |  |
| 200m FreeStyle (W) | Locked | 1994.0 |
| 200m Breast (W) | Progression |  |
| 200m Fly (W) | Locked | 1985.6 |
| 200m Back (W) | Locked | 1995.9 |
| 200m Individual Medley (W) | Progression |  |
| 400m Individual Medley (W) | Progression |  |
| 400m FreeStyle (W) | Locked | 1985.1 |
| 800m FreeStyle (W) | Locked | 1987.3 |
| 1500m FreeStyle (W) | Locked | 1981.2 |
| 4*100m FreeStyle Relay(W) | Progression |  |
| 4*100m Medley Relay (W) | Locked | 1988.8 |
| 4*200m FreeStyle Relay (W) | Progression |  |
| 50m FreeStyle (M) | Locked | 1989.5 |
| 100m FresStyle (M) | Locked |  |
| 100m Breast (M) | Progression |  |
| 100m Fly (M) | Locked | 1991.0 |
| 100m Back (M) | Progression |  |
| 200m FreeStyle (M) | Progression |  |
| 200m Breast (M) | Progression |  |
| 200m Fly (M) | Locked | 1988.4 |
| 200m Back (M) | Progression |  |
| 200m Individual Medley (M) | Progression |  |
| 400m Individual Medley (M) | Progression | 1998.3 |
| 800m FreeStyle (M) | Locked | 1987.9 |
| 1500m FreeStyle (M) | Locked | 1991.9 |
| 4*100m FreeStyle (M) | Progression | 1995.4 |
| 4*200m FreeStyle (M) | Locked | 1989.5 |
| 4*100m Medley Relay (M) | Progression |  |

**Supporting Table S1.** Evolution of the 10 best performers in all 70 events. Women events (W) and Men events (M) are sorted by disciplines (T&F vs. Swimming), and distances / type of efforts for T&F. All Swimming events show progression after the MRCOI.
