# Supplementary material for: Athlete Atypicity on the Edge of Human Achievement: Performances Stagnate after the Last Peak, in 1988
Source: PLoS One. 2010 Jan 20;5(1):e8800. doi: 10.1371/journal.pone.0008800 (PMC2808355; doi:10.1371/journal.pone.0008800)
Supplement: Materials and Methods S1 — Model and descriptors (d1-d3) description. Description of the model and the Gompertz function. Description of the three statistical descriptors d1-d3. (0.08 MB DOC) [file pone.0008800.s002.doc]

### **Supplementary Materials and Methods**

We studied performances evolution in T&F and swimming disciplines. For each event, only the best performance of an athlete was kept every year: any given athlete only appears once a year. We decided not to consider years where less than 4 performers were collected (this case appears for only 1 year in 70 events).

Thirty T&F events started before 1980 and were included. Javelin throws, pentathlon and decathlon were excluded because of major rules alterations. Relays and race walking were not present in the collected database.

Swimming data were collected from 1962 to 2008; only the years 1982 to 2008 present a complete dataset for all events resulting in the assessment of $A(t)$ over this period.

**(a) *Growth law of the first 10 world performers***

**(i) *Model description***

Growth laws were established on all events. Data were fitted for the following periods: 1891 to 1914, 1914 to 1945 and from 1945 to 2008 in T&F. For 6 sprint events (100m, 200m races for men and women and 400m, 110m hurdles race for men), an additional period was introduced after 1975 when automatic chronometers were introduced. Imprecision of previous chronometric instruments resulted in several performers having the same performances.

Performances are fitted for each period by a Gompertz function:

$$\hat{y}(t)=\left[\Delta.e^{a}\right].e^{-e^{b}\times e^{e^{c}.p(t')}}+d$$

With *p(t)* the vector of performances at year *t*, which was uniformized between [0; 1] using a linear transformation ($t\to t'$ [1]). $\Delta$ is a signed event indicator for the studied event; it is positive ($\Delta=+1$) for the timed chronometric events (with decreasing performances values) and negative ($\Delta=-1$) for the non-chronometric timed ones (increasing performances values). We used the exponential function to constrain Parameters *a, b, c, d* to $R_{+}^{*}$.

**(ii) *Analysis of Residuals***

An analysis of the residuals was performed to identify the years when performers are outlying the model. For each year of an event, the residuals of the model were expressed in percent of observed values:

where *p_i_(t)* is the *i-th* value of *p(t)*, *m* the last value of the vector (m ≤ 10) and $\hat{y}(t)$ is the estimated performance. Average value was computed every year for all events in both disciplines.

**(b) *Descriptive statistics***

The mean yearly coefficient of variation $\overline{cv(t)}$ determined the dispersion of the 10 best performers for each event and was defined by:

$$cv(t)=\frac{\sigma_{p(t)}}{\overline{p(t)}}$$

where$\sigma_{p(t)}$ is the standard deviation of *p(t)* and $\overline{p(t)}$ is the mean value of *p(t).* Mean of $cv(t)$ : $\overline{cv(t)}$ was computed for all events of each year.

The relative improvement $\kappa_{t}$ [1] between the means of the ten performers values ($\overline{p(t)}$) at year *t* and *t-1* were computed in order to show 1. the impact of the World Wars and 2. the yearly progression rate of performances. The average $\overline{\kappa_{t}}$value for all years of all events is computed and cumulated $\overline{\kappa_{t}}$is given (Fig. 2C). $\overline{\kappa_{t}}$ is used to show the Olympic Periodicity (Fig. 2D).

The Most Recent Change Of Incline (MRCOI) was given for swimming events: we performed a chronological iterative linear methodology between the first year and 2008 for each event.

**(c) *Characterizing Best Performers***

We analyzed the two disciplines separately. BP were selected from 1891 to 2008 for T&F and from 1982 to 2008 for swimming. Three specific “descriptors” (*d_1_-d_3_*) were then established, based on 3 measures that provide a score of the BP atypicity every year.

The first descriptor *d_1_* is the yearly distance of the BP to each other performer. For each event, let *p_1_(t)* be the studied BP at year *t*, and $E(t)$ the subset $E(t)=\left\{p_{1}(t)\notin p(t)\right\}$. The *i-th* performance of $E(t)$is noted $E(t)_{i}$ and *d_1_* is defined by:

$d_{1}(t)=\sum_{i=2}^{m} \frac{E(t)_{i}-p_{1}(t)}{p_{1}(t)}$ for chronometric events (decreasing curves)

$d_{1}(t)=\sum_{i=2}^{m} \frac{p_{1}(t)-E(t)_{i}}{p_{1}(t)}$for non-chronometric events (increasing curves)

The second one *d_2_* was the “durability” of a BP over the years before it is beaten by another performance:

$d_{2}(t)=\sum_{i=t+1}^{\beta} 1_{\left\{p_{1}(t)<p_{1}(i)\right\}}$ for chronometric events

$d_{2}(t)=\sum_{i=t+1}^{\beta} 1_{\left\{p_{1}(t)>p_{1}(i)\right\}}$ for non-chronometric events

Where $\beta$ is the year when *p_1_(t)* is ‘beaten’. This descriptor is time related, implying that a number of values will be altered as time goes on. Thus, newly performances are underestimated, as well as actual WR. $T$ defines the number of BP that use the date of ‘today’ as the temporal reference to assess *d_2_* (i.e. “actuals”, unbeaten performances). This side effect is time dependent, implying today’s marks will see their values corrected when beaten.

The third descriptor *d_3_* characterized the weight of each BP over all other performances for each event during the Olympic era. All performances of all events were centered by year in order to remove time trend:

$$p'(t)=p(t)-median(p(t))$$

Let *P* be the vector of all performances for all years for each event and *P’* the centered value of *P*. We identified the impact of each *p’_1_(t)* over all performances every year for each event:

$$d_{3}(t)=1-\frac{\sigma\left\{P'\right\}}{\sigma\left\{p'_{1}(t)\notin P'\right\}}$$

Where $\sigma\left\{P'\right\}$ is the standard deviation of *P’* for an event and $\sigma\left\{p'_{1}(t)\notin P'\right\}$ the standard deviation of the subset *P’* including *p’_1_(t)*.

The highest 5% values of each descriptor are selected and studied.

Descriptors *d_1_*, *d_2_*, *d_3_* were uniformized between [0; 1] to prevent weight effects. The Euclidian distance to the origin for each BP in the space of uniformized descriptors (Supporting Fig. S7) was thus given by:

$$A(t)=d_{1}(t)+d_{2}(t)+d_{3}(t)$$

and was computed for all *p_1_(t)* in all events regarding each discipline.

Each year, the mean value of $A(t)$ for all events is $\overline{A(t)}$.
