# Supplementary figures and images for: Athlete Atypicity on the Edge of Human Achievement: Performances Stagnate after the Last Peak, in 1988
Source: PLoS One. 2010 Jan 20;5(1):e8800. doi: 10.1371/journal.pone.0008800 (PMC2808355; doi:10.1371/journal.pone.0008800)

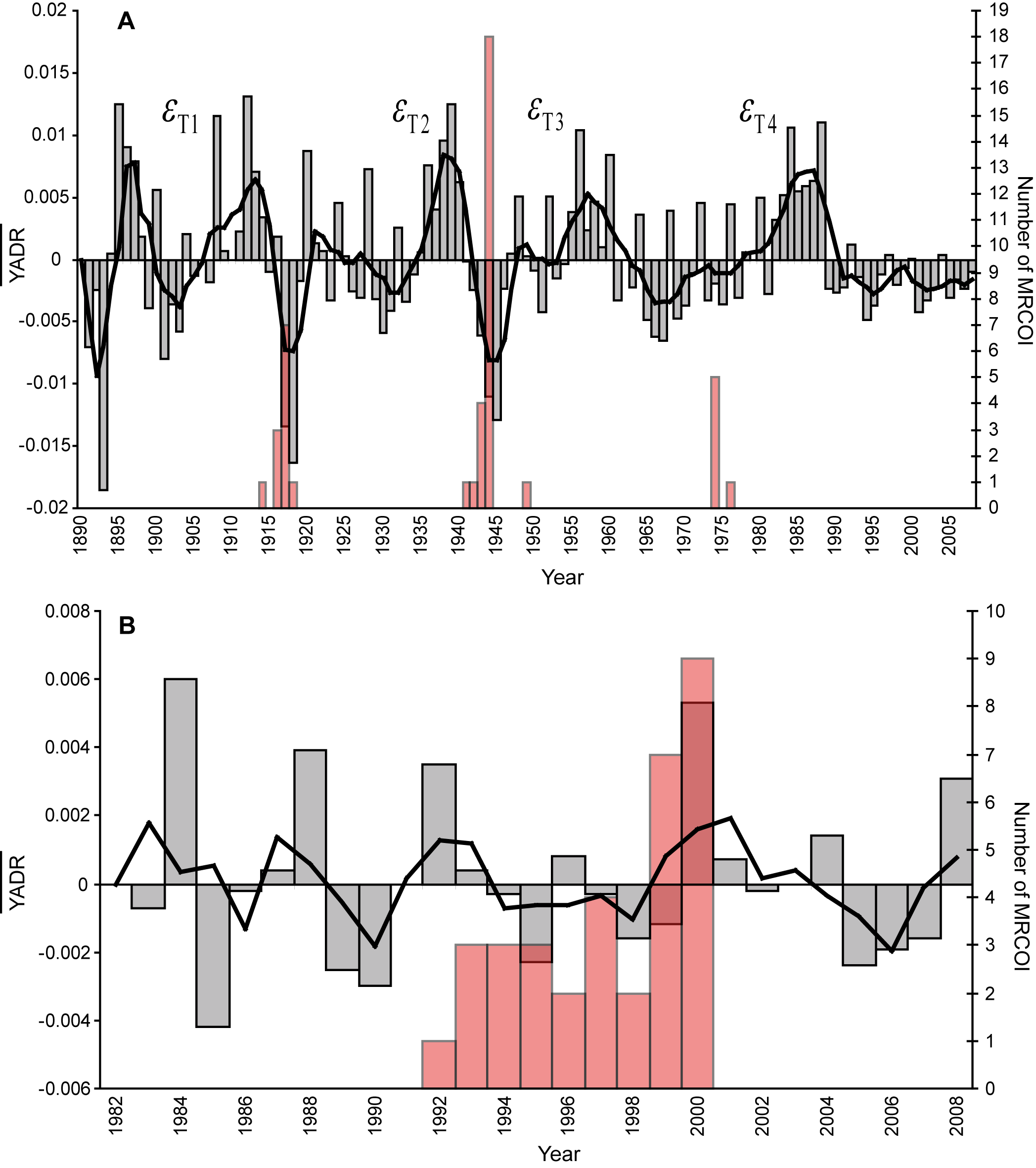

Supplement: Figure S1 — Secular evolution of the yearly average distance of residuals (YADR). A. Secular evolution of YADR in T&F (gray bars, left ordinate) with the number of changes of inclines (red bars, right ordinate). B. Secular evolution of YADR in swimming (gray bars, left ordinate) with the number of MRCOI detected each year (red bars, right ordinate). The analysis of residuals of T&F events (A) presents several stages: two major deviations periods (Et1,Et2) consecutive to WWs and two more recent peaks (Et3,Et4) including the largest one (Et4). After the initial era (1890 to 1900), high positive deviations from the model are spotted during the following periods: 1908 to 1915 (Et1, peak in 1912: 1.31%), 1935 to 1942 (Et2, peak in 1939: 1.25%), 1953 to 1963 (Et3, peak in 1956: 1.04%), 1977 to 1991 (Et4, peak in 1988: 1.1%). Swimming YADR (B) presents numerous variations of low amplitudes (peak in 1984 at 0.6%). The major number of MRCOI detected in swimming is spotted in 1999 and 2000, contemporary to the introduction of swimsuits, allowed by the FINA in 1999. (0.85 MB TIF) [file pone.0008800.s003.tif]

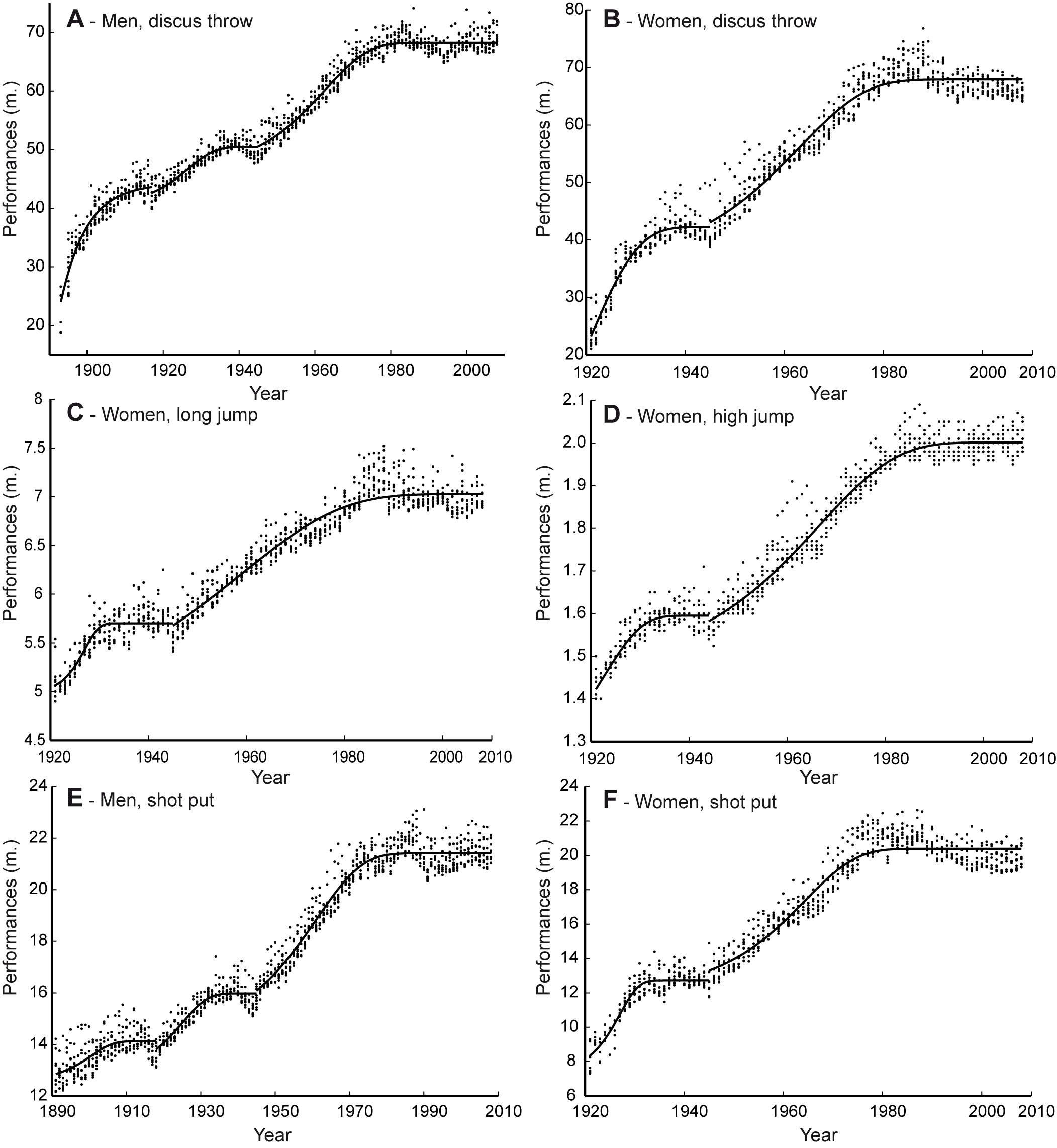

Supplement: Figure S2 — Model fitting on 6 T&F. A. Men discus throw (T&F) (R2i = 0.85; R2ii = 0.83; R2iii = 0.94), “halted” in 1984.2. B. Women discus throw (T&F) (R2i = 0.88; R2ii = 0.92), “halted” in 1988.4. C. Women long jump (R2i = 0.70; R2ii = 0.90), “halted” in 1996.8. D. Women high jump (R2i = 0.78; R2ii = 0.95), “halted” in 1995.2. E. Men shot put (R2i = 0.43; R2ii = 0.80; R2iii = 0.93), “halted” in 1983.9. F. Women shot put (R2i = 0.86; R2ii = 0.90), “halted” in 1983.9. (0.68 MB TIF) [file pone.0008800.s004.tif]

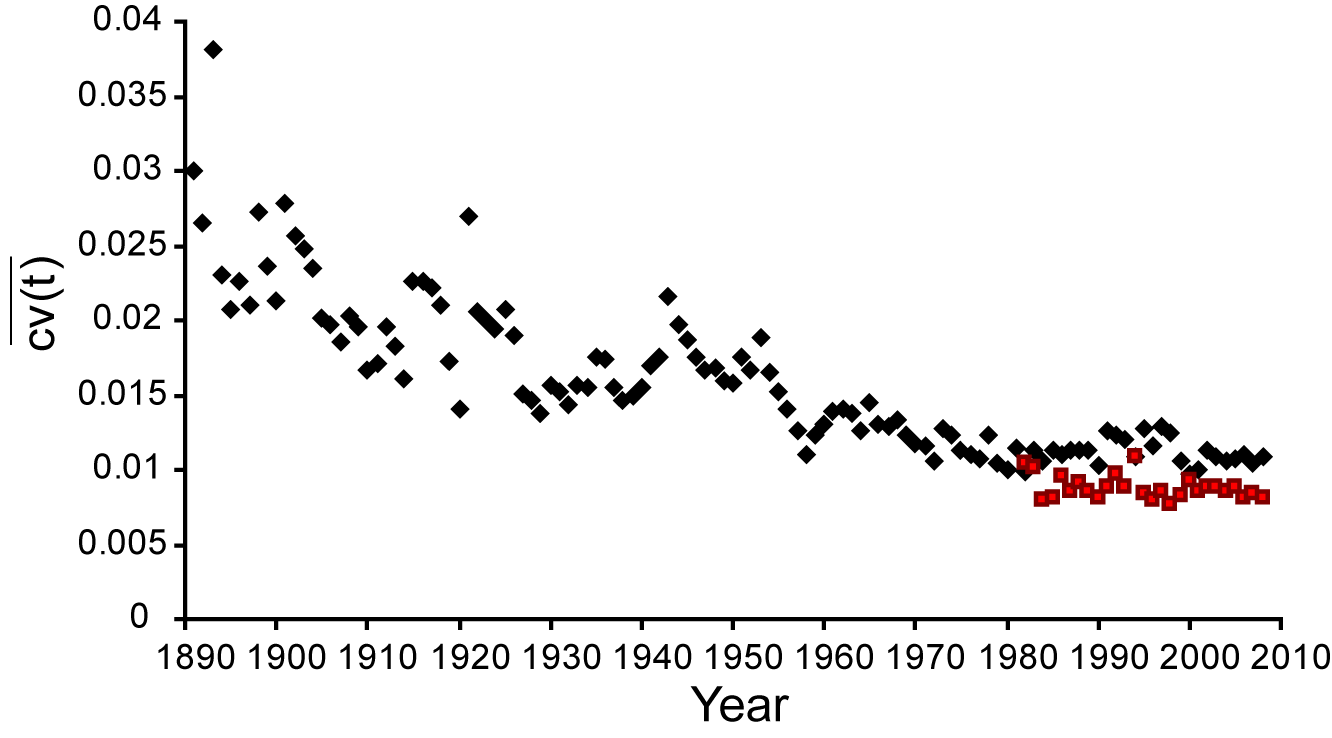

Supplement: Figure S3 — Evolution of cv(t) factor in T&F and swimming. cv(t) values are decreasing over time in T&F (black dots); cv(t) values show no trend in swimming (red squares) on a shorter follow-up period. T&F cv(t) evolution is linked to historic events: after World War II values are getting tighter (<0.015) suggesting all performances are progressing in the same range. (0.16 MB TIF) [file pone.0008800.s005.tif]

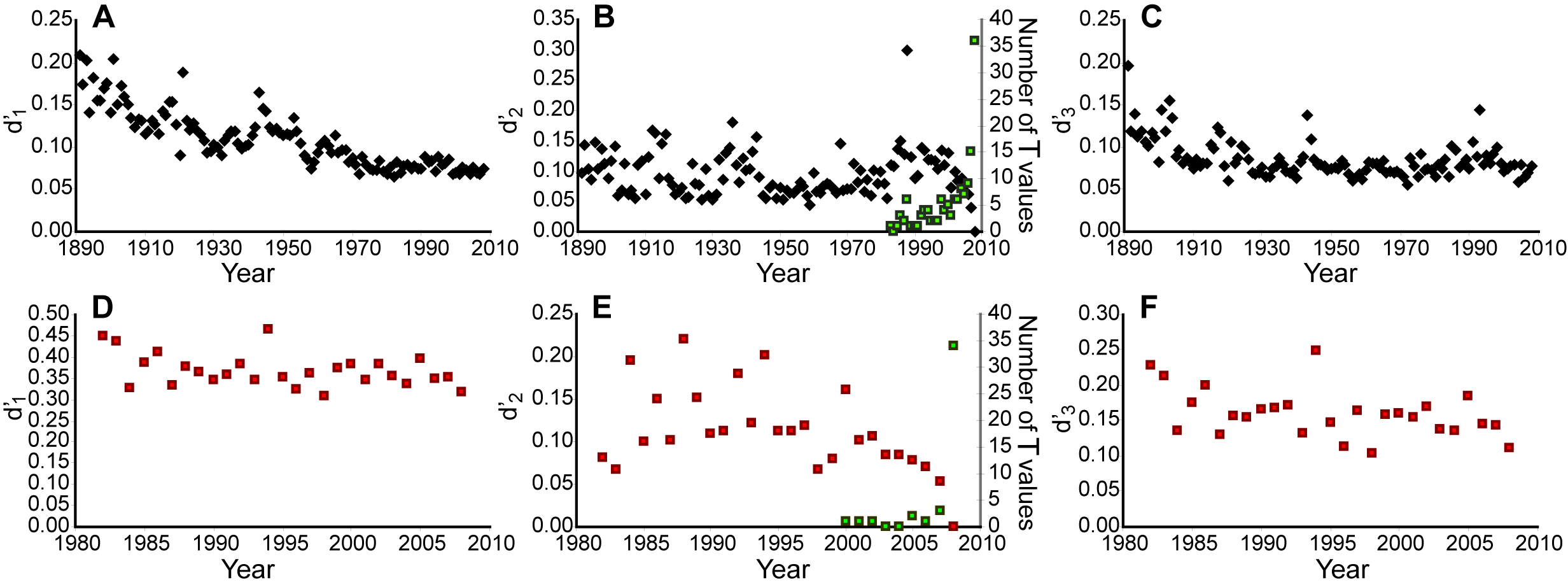

Supplement: Figure S4 — Secular evolution of descriptors. Evolution is given in T&F (A, B, C, black dots) and swimming (D, E, F, red squares). Descriptor d2 presents a decreasing evolution from 2003 to 2008, which is related to time. It is assessed by T, measuring the number of performances still unbeaten (green squares) in both disciplines. High average values of T&F descriptors are spotted at d2: 1988, d3: 1993. (0.37 MB TIF) [file pone.0008800.s006.tif]

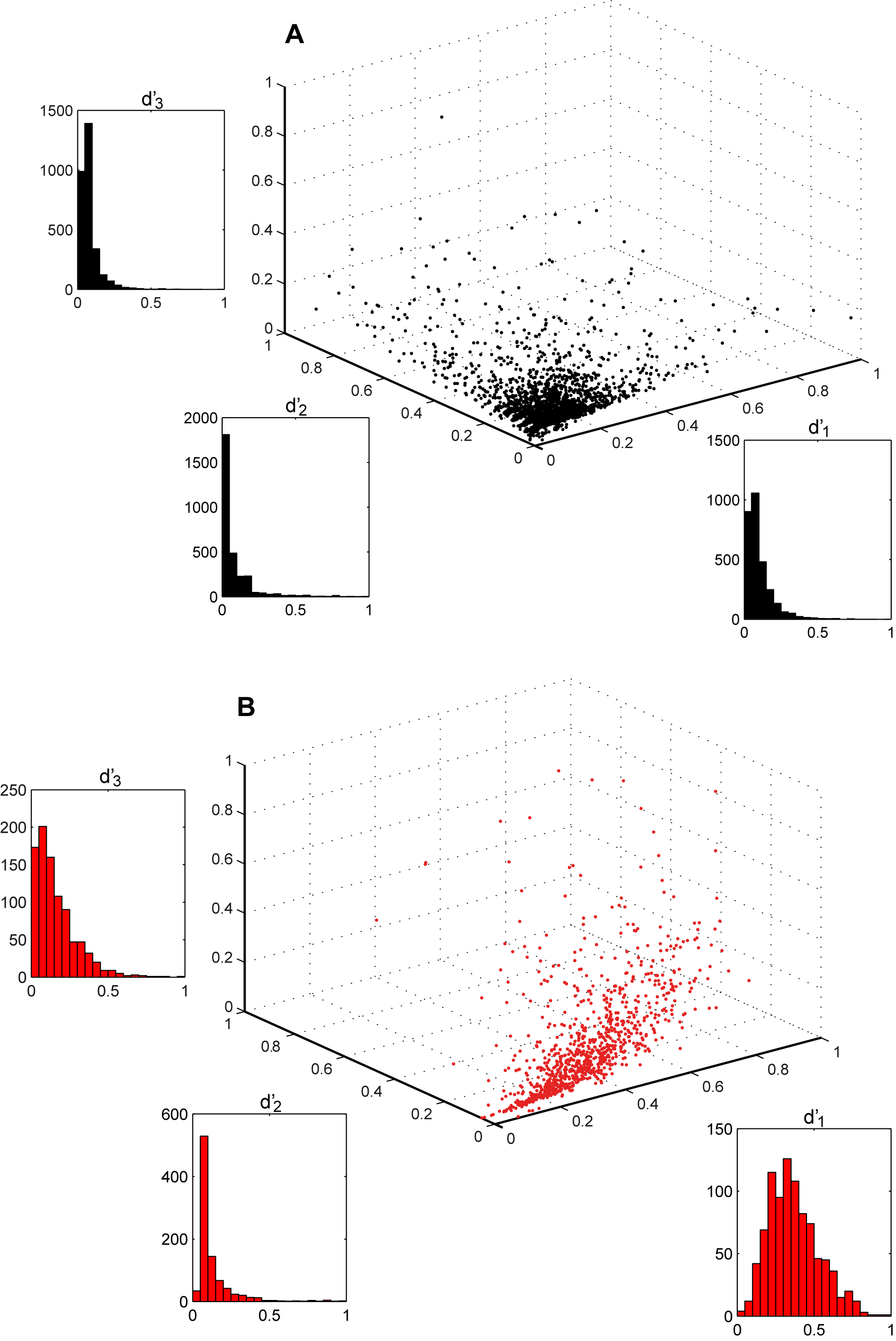

Supplement: Figure S5 — Spatial repartition and distributions of the 3 uniformized descriptors d1, d2, d3. Spatial repartition and distributions of the 3 uniformized descriptors d1, d2, d3 for T&F (A, black dots and bars) and swimming (B, red dots and bars). All distributions are unimodal with positive skew. For T&F: d'1 skew = 2.9; d'2 skew = 3.8; d'3 skew = 4.5; for swimming: d'1 skew = 0.6; d'2 skew = 3.6; d'3 skew = 1.7; p-value<0.001 for all (d'Agostino Skewness test, alternative hypothesis: positive skewness). Outliers are located in right tails. (0.61 MB TIF) [file pone.0008800.s007.tif]
